# Acetyl-lupeolic acid inhibits Akt signaling and induces apoptosis in chemoresistant prostate cancer cells *in vitro* and *in vivo*

**DOI:** 10.18632/oncotarget.19101

**Published:** 2017-07-08

**Authors:** Claudia Schmidt, Cornelia Loos, Lu Jin, Michael Schmiech, Christoph Q. Schmidt, Menna El Gaafary, Tatiana Syrovets, Thomas Simmet

**Affiliations:** ^1^ Institute of Pharmacology of Natural Products and Clinical Pharmacology, Ulm University, Ulm, Germany; ^2^ Present address: Rommelag CMO, Sulzbach-Laufen, Germany; ^3^ Present address: Institute of Protein Biochemistry, Ulm University, Ulm, Germany; ^4^ Present address: Department of Pharmacognosy, College of Pharmacy, Cairo University, Giza, Egypt

**Keywords:** triterpenoids, apoptosis, prostate cancer, allosteric Akt inhibitor

## Abstract

The triterpenoid acetyl-lupeolic acid (ac-LA) isolated from the oleogum resin of *Boswellia carterii* reduced the viability of a panel of cancer cell lines more efficiently than lupeol. There was no detectable intracellular conversion of ac-LA to lupeol and *vice versa*. In contrast to docetaxel, ac-LA did not induce selection of treatment-resistant cancer cells. By various parameters including DNA fragmentation, ac-LA was shown to induce apoptosis in androgen-independent PC-3 cells, whereas in MDA-MB-231 breast cancer cells, ac-LA led to cell accumulation in the G_2_/M phase of the cell cycle, but not to apoptosis. *In silico* docking combined with *in vitro* kinase assays implied that ac LA potently inhibits Akt mainly by direct binding to the pleckstrin homology domain. Consistently, an Akt1 mutant deficient of the PH domain afforded partial resistance to ac-LA and complete resistance to lupeol and the Akt inhibitor III. Ac-LA inhibited phosphorylation of downstream targets of the Akt signaling pathway, which was followed by inhibition of the mTOR target p70 ribosomal six protein kinase and the nuclear accumulation of p65/NF-κB, β-catenin, and c-myc, as well as loss of the mitochondrial membrane potential. Ac-LA exhibited antiproliferative, proapoptotic, and antitumorigenic effects on PC-3-tumors xenografted either on chick chorioallantoic membranes or in nude mice. Ac-LA exhibited a clearly better safety profile than docetaxel or lupeol during chronic administration *in vivo*. In contrast to lupeol, ac-LA also inhibited release of vascular endothelial growth factor *in vitro* and accordingly angiogenesis *in vivo*. Thus, ac-LA deserves further exploration as a potential new antitumor compound.

## INTRODUCTION

In many Western countries, prostate cancer is the most common cancer in men. With an adequate and radical therapy, the prognosis for localized prostate cancer is often good. However, when tumors relapse, they often exhibit an incurable androgen-independent phenotype associated with bone metastasis. Under those circumstances, treatment modalities become scarce and prognosis worsens dramatically [1]. Docetaxel-based chemotherapy was the first exhibiting some, though only very limited, survival advantage for these patients [1]. Likewise, for pancreatic cancer, the five-year relative survival of patients is very low and ranges between 2 and 8%. Whereas only 3% of cancer incidences in the United States are pancreatic cancer, it is the fourth leading cause of cancer-related deaths. The life expectancy of patients with metastatic disease is merely 3–5 months [2]. Hence, for both cancer types, there are no effective treatment regimens, which would improve prognosis [1, 2]. The development of new effective chemotherapeutic agents is therefore indispensable.

Activation of the serine/threonine kinase Akt/PKB is involved in the regulation of many cellular processes including cell survival, cell cycle progression, proliferation, differentiation, metabolism, migration and angiogenesis. Accordingly, dysregulation of the PI3K/Akt pathway plays a central role in the development and progression of human cancers [1]. In prostate cancer, Akt is frequently constitutively active due to the loss of the negative regulator phosphatase and tensin homolog (PTEN) or as a result of Akt mutations. Thus, about 42% of primary prostate cancer and 100% of metastatic tumors exhibit mutations leading to PI3K/Akt activation [1].

Akt is composed of an N-terminal pleckstrin homology (PH) domain, a catalytic domain, and a C-terminal regulatory domain. Following the activation of phosphatidylinositol-3-kinase (PI3K) by growth factor receptors, the resulting second messenger phosphatidylinositol 3,4,5-trisphosphate (PIP3) binds to the PH domain of proteins such as Akt or 3-phosphoinositide-dependent kinase 1 (PDK1) and recruits them to the plasma membrane. This binding leads to a conformational change and activation of Akt by PDK1 and the mammalian target of rapamycin complex 2 (mTORC2) by phosphorylation on two residues. Phosphorylated Akt dissociates from the plasma membrane to regulate multiple pathways [3]. The tumor suppressor PTEN is an important negative regulator of PDK1 and Akt acting by dephosphorylation and the reduction of PIP3 levels [3].

One function of Akt is the activation of the mammalian target of rapamycin complex 1 (mTORC1), a critical positive regulator of P70 ribosomal six protein kinase (P70S6K). P70S6K is an activator of the protein synthesis machinery resulting in increased protein synthesis and cell proliferation. The significance of this pathway can be deduced from the fact that it controls synthesis of angiogenic factors such as vascular endothelial growth factor (VEGF), which promotes tumor development and metastasis [4]. Besides, Akt activates transcription factors such as c-myc or β-catenin regulating cell growth and proliferation by inhibitory phosphorylation of their negative regulator, glycogen synthase kinase 3 (GSK-3). Additionally, Akt influences cell survival by activation of the transcription factor nuclear factor of κB (NF-κB) *via* activation of IκB kinase (IKK) resulting in degradation of the NF-κB inhibitor IκBα.

The diversity of Akt effects is in part due to different functions of the three Akt isoforms, Akt1, Akt2, and Akt3 [3]. The Akt isoforms exhibit some redundancy of their biological functions, but also demonstrate some specificity. Thus, Akt1 deficiency is characterized by early mortality and growth retardation due to defective placental development, whereas an Akt2 knockout is characterized by the development of severe diabetes because AKT2 increases glucose uptake by promoting cellular membrane localization of the glucose transporter isoform 4 [3]. The role of different Akt isoforms in cancer development is still to be unraveled. Some evidence points to a particular role of constitutive Akt1 activation in tumor promotion. Thus, mice with mammary gland-specific AKT1 expression that are systemically treated with the carcinogen DMBA develop breast cancer. Similarly, mutations of Akt1, but not of Akt2 or Akt3 genes are significantly increased in a number of human cancers, although the clinical significance of those mutations is still to be established [3]. The androgen-independent prostate cancer cell line PC-3 expresses only Akt1 and Akt2 [5]. Due to the cellular processes involved, therapeutic targeting of Akt-signaling may harbor substantial potential for the discovery of new chemotherapeutic agents [1, 3].

Despite significant achievements in the development of synthetic small-molecule library testing, *in silico* modeling, and rational pharmacophore design, natural products still play an important role in drug discovery. Indeed, particularly in cancer therapy, about 80% of clinically used agents are inspired or directly derived from natural products [6]. Among them, triterpenoids are of particular importance due to their versatile biological activities [7]. Lupeol is one of the better explored plant-derived triterpenes, which has been shown to exhibit anti-inflammatory, antiproliferative, and antitumor activities by selectively targeting tumor cells [8–10]. Although, lupeol was reported to exhibit low acute systemic toxicity, its antiproliferative and cytotoxic effects on tumor cells occurring at concentrations as high as 50–800 μM [11, 12] indicates, that the necessary plasma concentrations will be hardly achieved in clinical settings and lupeol derivatives with higher potency would be preferable.

Oleogum resins of *Boswellia* species, commonly named frankincense, contain a plethora of various pentacyclic triterpenes with anti-inflammatory and anticancer properties [5, 7, 13–21]. In addition to lupeol, we have isolated the novel pentacyclic triterpenoid, acetyl-lupeolic acid (3α-acetyl-lup-20(29)-en-24-oic acid; ac-LA) from the oleogum resin of *Boswellia carterii* [16, 22]. In order to analyze the efficacy of this new triterpenoid in the treatment of chemoresistant tumors, we compared the pharmacological properties of ac-LA to lupeol *in vitro* by using a panel of human cancer cell lines and *in vivo* by using pre-established prostate cancers xenografted onto the chick chorioallantoic membrane (CAM) and in mice. We have also delineated a molecular mechanisms and intracellular targets of ac-LA in cancer cells. We show, that ac-LA exhibits a better safety profile and higher therapeutic efficacy compared to lupeol.

## RESULTS

### Stability of ac-LA upon uptake by prostate cancer cells

**Figure 1:**
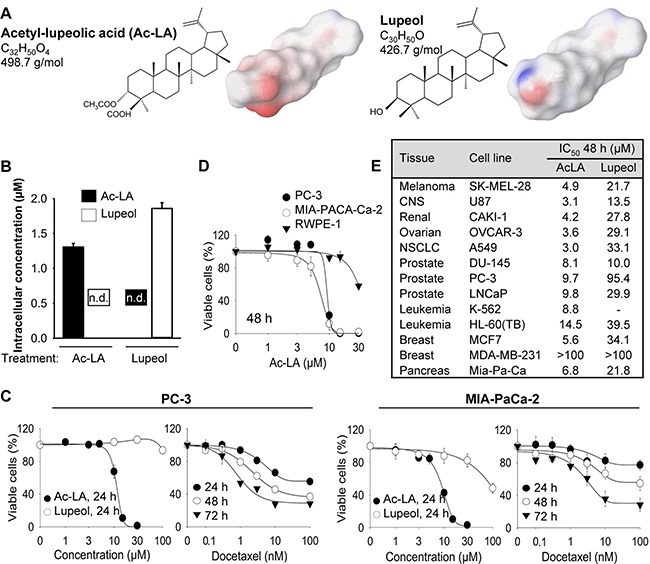
Ac-LA selectively inhibits viability of treatment-resistant cancer cell lines (**A**) Chemical structures of ac-LA and lupeol and surface charge distribution. Atoms with negative partial charge are colored red, the atoms with positive partial charge are colored blue. (**B**) Ac-LA is not converted to lupeol in the prostate cancer cell line PC-3. Cells take up ac-LA and lupeol to a similar extent, no intracellular conversion of ac-LA into lupeol and *vice versa* could be detected within 6 h. Cells were treated with ac-LA and lupeol (10 μM each) and analyzed by reverse-phase HPLC. (**C**) In comparison to lupeol, ac-LA exhibits higher time- and concentration-dependent inhibition of viability of prostate and pancreatic cancer cells and in contrast to docetaxel does not induce resistance. Cells were treated for 24-72 h and viability was analyzed by XTT assay. (**D**) Nontumorigenic prostate epithelial RWPE-1 cells are relatively resistant to treatment with ac-LA. MIA-PaCa-2, PC-3, and RWPE-1 cells were treated for 48 h and cell viability was analyzed by XTT. (**E**) Ac-LA inhibits viability of a panel of cancer cell lines more potently than lupeol. Cells were treated and analyzed as in D. All results are mean ± SEM of three independent experiments each performed in triplicate.

The molecular structure of ac-LA is similar to that of the better explored lupeol (Figure 1A). However, due to an acetyl and a carboxyl group at the first ring, the ac-LA molecule exhibits a much more negative surface charge compared to lupeol. Lupeol has been reported to exhibit various favorable pharmacotherapeutic effects including anti-inflammatory and anticancer activity [9]. Therefore, it was important to ensure that there is no intracellular conversion of ac-LA to lupeol and *vice versa*, which could obscure the results. As analyzed by reverse-phase HPLC, six hours after addition to prostate cancer cells, the intracellular concentrations of lupeol and ac-LA were not significantly different 1.86 ± 0.08 μM and 1.31 ± 0.05 μM, respectively). Similarly, there was neither conversion ac-LA to lupeol nor lupeol to ac-LA (Figure 1B).

### *In vitro*, ac-LA is more cytotoxic to cancer cells than lupeol

The viability of the androgen-insensitive prostate cancer cell line PC-3 or the pancreatic cancer cell line MIA-PaCa-2 was concentration- and time-dependently reduced by the treatment with ac-LA (Figure 1C). The IC_50_ values for ac-LA in PC-3 cells was 11.3 ± 0.6 μM and 7.2 ± 0.1 μM for the 24 h and 72 h treatment, respectively. MIA-PaCa-2 cells were slightly more sensitive to ac-LA with IC_50_ values of 8.9 ± 0.3 μM and 6.2 ± 0.4 μM after 24 h and 72 h treatment, respectively. In contrast, in both cell lines, lupeol was less efficient with IC_50_ values of > 100 μM for the 24 h-treatment of PC-3 cells and of 91.6 ± 6.8 μM for the 24 h-treatment of MIA-PaCa-2 cells. Interestingly, the non-tumorigenic prostate epithelial cells RWPE-1 were relatively resistant to ac-LA indicating specificity for cancer cells (Figure 1D). Although the cytotoxic effect of the commonly used chemotherapeutic docetaxel was in the nM range, a substantial number of PC-3 and MIA-PaCa-2 cells exhibited resistance to docetaxel and retained viable even at concentrations > 1 μM (Figure 1D and data not shown). Thus, after 72 h about 28.3 ± 1.0% of PC-3 and 28.4 ± 7.9% of MIA-PaCa-2 cells exhibited resistance to 100 nM docetaxel. In contrast, no resistant cancer cells were observed after treatment with ac-LA (Figure 1C, 1D).

We further compared the effects of ac-LA and lupeol on a panel of 11 cancer cell lines of melanoma, CNS, ovary, NSCLC, leukemia, prostate, and mammary gland origin included by the US National Cancer Institute (NCI) into the human tumor cell line anticancer drug screen panel (NCI60, https://dtp.cancer.gov) (Figure 1E). In all cell lines analyzed, ac-LA exhibited superior antiproliferative activity compared to lupeol. Thus, ac-LA inhibited viability of all three prostate cancer cell lines with similar efficacy. It was even more potent against melanoma, CNS, ovary, NSCLC, and leukemia cells. Unexpectedly, the triple-negative (ER^-^, PR^-^, HER2^-^) breast cancer cell line MDA-MB-231, but not the hormone-sensitive breast cancer cells MCF7, exhibited high resistance to ac-LA. Interestingly, there was a correlation between ac-LA and lupeol as to their ability to inhibit the viability of different cell lines (Pearson's test, *R* = 0.927, *p* = 0.0001, *n* = 10, Systat Software Inc., San Jose, CA), which indicates that they may have similar molecular targets.

To unravel the mechanisms of the antiproliferative activity of ac-LA, we have further analyzed effects of ac-LA and lupeol on sensitive prostate PC-3 and resistant breast MDA-MB-231 cell lines.

### Ac-LA induces apoptosis in PC-3 cells

**Figure 2:**
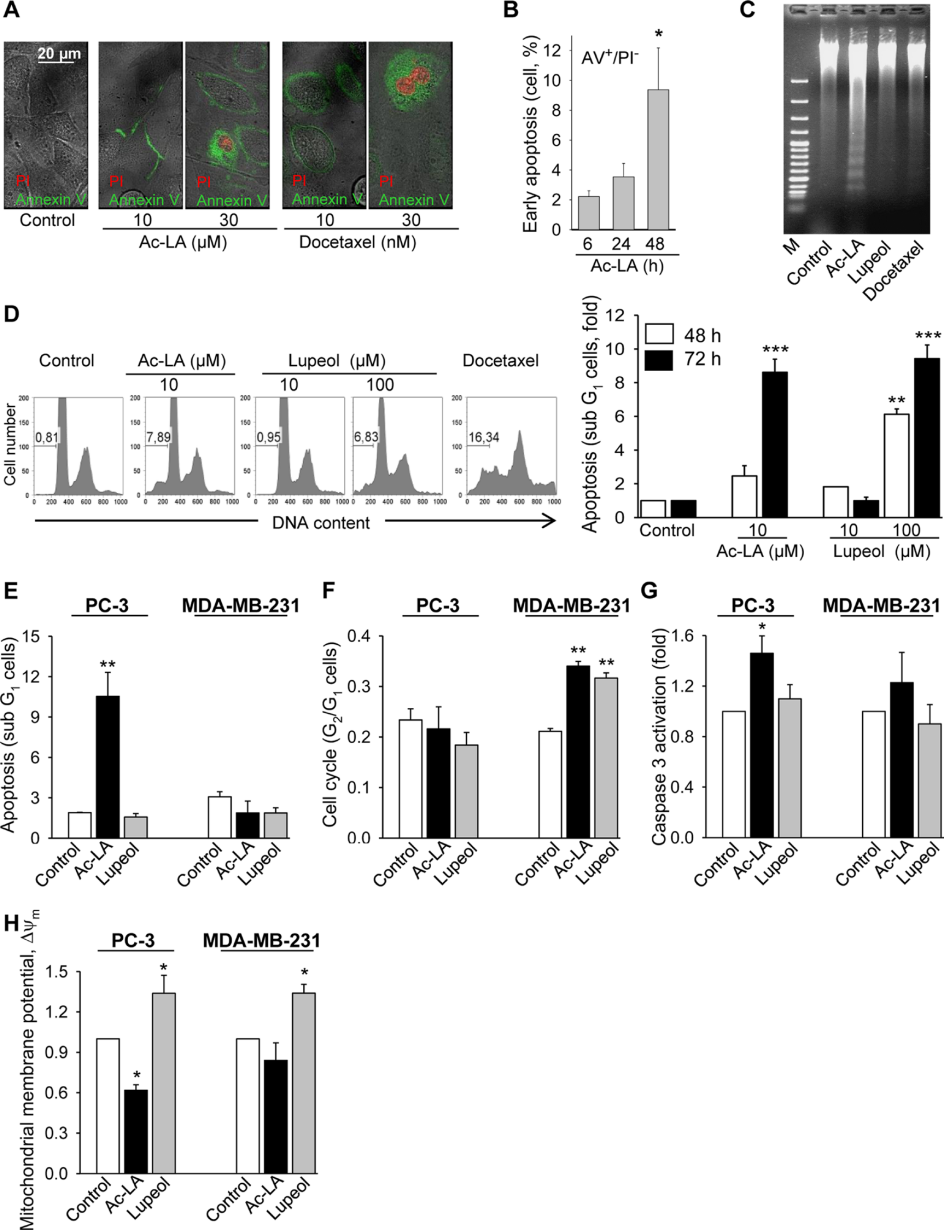
Ac-LA induces apoptosis in prostate cancer cells (**A**) Ac-LA induced translocation of phosphatidylserine to the outer leaflet of the cell membrane. PC-3 cells were treated for 24 h, stained with annexin V-FITC (green) and PI (red), and analyzed by confocal fluorescence microscopy. (**B**) PC-3 cells were treated for different time periods with 10 μM ac-LA, stained as in A and analyzed by FACS. (**C**) Ac-LA induces DNA laddering in PC-3 cells treated for 48 h with 10 μM ac-LA; 100 μM lupeol and 10 nM docetaxel were used for comparison. (**D**) Ac-LA increases the apoptotic sub-G_1_ cell population. Left panel: typical histograms are shown (treatment for 72 h). (**E**) Ac-LA induces apoptosis in prostate PC-3 cancer cells more efficiently than in the breast cancer cell line MDA-MB-231. Cell were analyzed as in D after treatment with 10 μM of either ac-LA or lupeol for 72 h. (**F**) Cell-cycle analysis of cells treated with ac-LA or lupeol as in E. (**G**) Caspase 3 activation in PC-3 cells treated as in E for 6 h and analyzed using the caspase 3 substrate Z-DEVD-R110 followed by flow cytometry. (**H**) AcoA induces loss of mitochondrial membrane potential in PC-3 but not MDA-MB-231 cells. Cell were treated as in E for 6 h and the mitochondrial membrane potential, ΔΨm (red/green fluorescence intensity ratio), was analyzed using JC-1 dye staining followed by flow cytometry. All results are mean ± SEM, *p < 0.05, **p < 0.01, ***p < 0.001, *n* = 3.

Examination of several apoptosis parameters [23] revealed the induction of apoptosis by ac-LA in prostate cancer cells. As a common sign of apoptotic cells, phosphatidylserine normally located at the inner leaflet of the plasma membrane, was displayed on the outer cell surface after treatment with ac-LA or docetaxel (Figure 2A). To distinguish between early stages of apoptosis and late phase apoptosis or necrosis, cells were additionally stained with propidium iodide (PI), which enters the cells when the membrane loses its integrity; subsequent intercalation into DNA leads to a profound increase of PI fluorescence. The proportion of early apoptotic annexin V^+^/PI^-^ cells was time-dependently increased in PC-3 cells treated with ac-LA as measured by flow cytometry. After 48 h, 9.4 ± 2.8% of PC-3 cells were early apoptotic (Figure 2B).

During apoptosis, activation of cysteine proteases triggers destruction of cellular components and activation of endonucleases creating a specific DNA cleavage pattern of 180 bp and multiples. This DNA fragmentation was evident in ac-LA-treated PC-3 cells (Figure 2C). Induction of apoptosis by ac-LA was likewise confirmed by flow cytometric analysis of the PI-stained prostate cancer cells. Thus the apoptotic sub-G_1_ cell population was increased in the ac-LA treated PC-3 cells. Lupeol was about tenfold less potent compared to ac-LA (Figure 2D).

Comparative analysis of apoptotic parameters in PC-3 and MDA-MB-231 cells revealed that in PC-3 cells, ac-LA increases the sub-G_1_ cell population after 48 and 72 h (Figure 2E) and induces the caspase 3 activation as early as after 6 h of treatment (Figure 2G). At equimolar concentrations (10 μM), lupeol was unable to initiate apoptosis in PC-3 cells. In agreement with the analysis of cell viability (Figure 1E), apoptotic parameters were increased in MDA-MB-231 cells neither by ac-LA nor by lupeol (Figure 2E and 2G). Interestingly, both, ac-LA and lupeol, induced accumulation of MDA-MB-231 cells in the G_2_/M cell-cycle phase (Figure 2F), which might contribute to cell resistance to apoptosis.

Since early caspase activation might point to mitochondrial damage by ac-LA, changes in mitochondrial membrane potential were analyzed. In agreement with caspase 3 activation, within 6 h of exposure, ac-LA induced significant depolarization of the mitochondrial membrane in PC-3, but not in MDA-MB-231 cells. Interestingly, in both cell lines, lupeol induced hyperpolarization of the mitochondrial membrane, which indicates that mitochondria in both cell lines are capable of ATP production [24].

Taken together, ac-LA is a strong inducer of apoptosis in androgen-independent, treatment-resistant PC-3 prostate cancer cells, but not in triple-negative MDA-MB-231 breast cancer cells.

### Ac-La inhibits Akt-signaling

**Figure 3:**
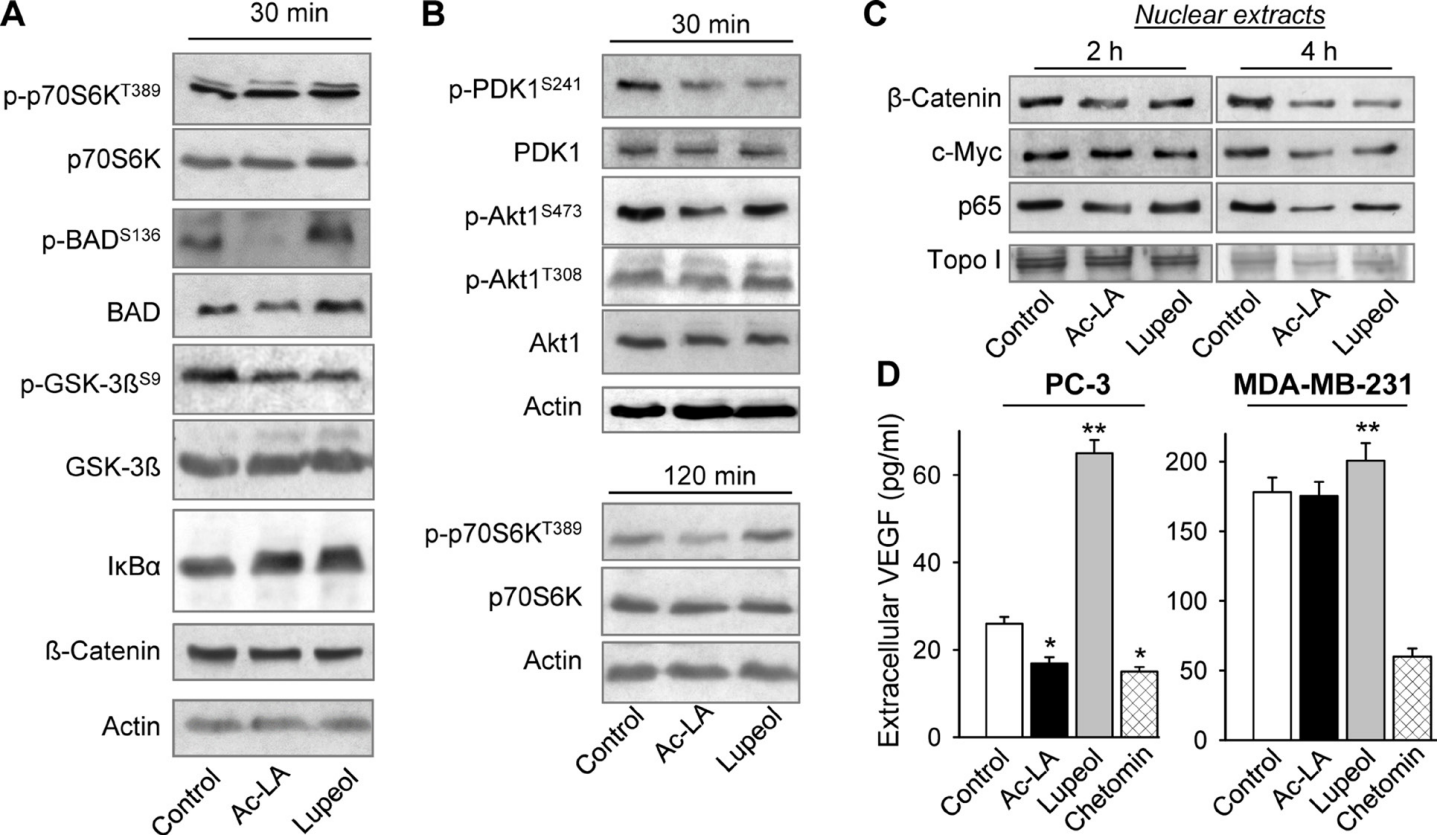
Ac-LA inhibits the Akt signaling pathway and the release of angiogenic VEGF in prostate cancer cells (**A**) Ac-LA rapidly inhibits the Akt, but not the mTOR downstream targets. Whole cell lysates of PC-3 cells treated with ac-LA or lupeol (each 10 μM) for 30 min were analyzed by using Western immunoblotting. Actin served as loading control.(**B**) Ac-LA rapidly inhibits phorphorylation of PDK1 and Akt, and later also that of the mTOR target p70S6K. Cells were treated and analyzed as in A. (**C**) Cells were treated as in A for 2 or 4 h, nuclear extracts were isolated and the Akt downstream targets were analyzed by using Western immunoblotting. (**D**) Cells were treated as in A for 24 h, supernatants were collected, and the VEGF contents were analyzed by ELISA. Chetomin (100 nM) was used as positive control. Results are mean ± SEM, *p < 0.05, **p < 0.01, ***p < 0.001, *n* = 3.

We have previously shown that different pentacyclic triterpenoids target diverse antiapoptotic pathways in cancer cells [5, 17, 18, 20, 21]. As early as 30 min after treatment, ac-LA inhibited phosphorylation of the downstream targets of Akt, GSK-3β, BAD, and increased the amount of cellular IκBα. At this time point, there were no changes in phosphorylation of the mTOR substrate p70S6K (Figure 3A), which became, however, apparent, when cells were treated with ac-LA for 120 min (Figure 3B). Thus, the inhibition of the Akt pathway preceded that of the mTOR pathway. By contrast, lupeol rather increased the phosphorylation of p70S6K at this time point. These data suggested that ac-LA might target Akt or PDK1, a kinase upstream of Akt. Indeed, ac-LA potently and rapidly inhibited activation of PDK1 and of its downstream target Akt1 (Figure 3B). Lupeol and ac-LA were equally efficient in inhibiting the PDK1 phosphorylation, but ac-LA was more efficient in inhibiting the Akt activation compared to lupeol.

As Akt controls activation of several transcription factors essential for cell survival and proliferation, we have further analyzed the expression of its downstream targets, β-catenin, c-myc, and p65/NF-κB in nuclear extracts. The concentrations of β-catenin and p65/NF-κB were reduced in nuclei of ac-LA- and lupeol-treated prostate cancer cells at 2 h and of all three proteins, 4 h after addition of the terpenoids (Figure 3C). Although we have used the same concentration of lupeol and ac-LA (10 μM), the lupeol effects were somewhat retarded compared to those of ac-LA (Figure 3A–3C).

Among other targets of the Akt/mTOR/p70S6K pathway is hypoxia-inducible transcription factor-1α, which increases expression of angiogenic factors like vascular endothelial growth factor (VEGF) or platelet-derived growth factor (PDGF) [4]. The VEGF production by PC-3 and MDA-MB-231 was effectively inhibited by the HIF-signaling inhibitor chetomin (Figure 3D). In agreement with the previous data demonstrating inhibition of the Akt/mTOR/p70S6K pathway by ac-LA, it significantly downregulated the production of VEGF by PC-3 cells. In PC-3 cells, treatment with lupeol at the same concentrations as ac-LA even significantly increased the VEGF release into the medium (Figure 3D). By contrast, the VEGF production by MDA-MB-231 cells remained largely unchanged by 10 μM ac-LA, or by equimolar concentrations of lupeol.

### Unraveling the role of the PH domain in the ac-LA-induced inhibition of Akt signaling

**Figure 4:**
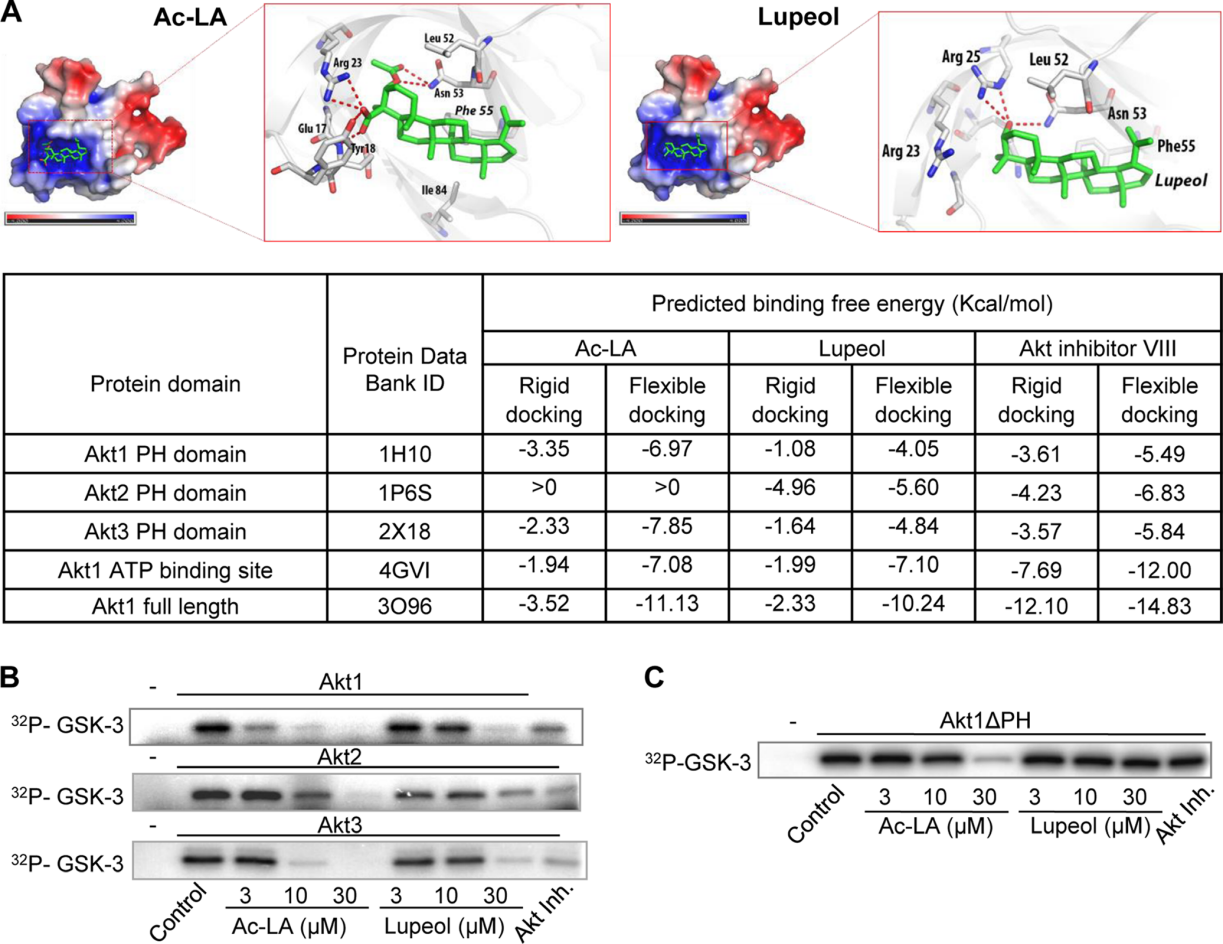
Ac-LA binds to the AKT PH domain and the ATP-binding site and inhibits the kinase activity of AKT (**A**) Predicted binding mode of ac-LA to the PH domain of Akt1 (PDB ID: 1H10, grey sticks: carbon, red sticks: oxygen, blue sticks: nitrogen, red dotted line: hydrogen bonds, atoms with negative partial charge are red, atoms with positive partial charge are blue). Ac-LA and lupeol are shown in green. Table shows the calculated binding energy of ac-LA to the PH domain and the ATP-binding site compared to that of lupeol and Akt inhibitor VIII. (**B**) Ac-LA inhibits the kinase activity of human recombinant Akt1, Akt2, and Akt3 more efficiently than lupeol. Akt inhibitor III (10 μM) was used as positive control, GSK-3 - substrate. (**C**) AKT1 lacking the PH domain (Akt1ΔPH) is less sensitive to ac-LA as analyzed in a kinase assay using GSK-3 as substrate. ^32^P-labelled substrate in (B and C) was scanned using a PhosphorImager. All data are representative of at least three independent experiments.

To analyze whether ac-LA might bind to and thus inhibit Akt directly, we have chosen two approaches, computational modeling and an *in vitro* kinase assay. *In silico* docking was done with all three Akt isoforms using known crystal structures. In addition, we have performed docking of ac-LA to the ATP-binding domain of Akt. Docking was performed with Ac-LA, lupeol, and Akt inhibitor VIII. In agreement with the *in vitro* data, molecular docking predicted a high affinity binding of ac-LA to a positively charged binding pocket of the Akt1 PH domain (residues 1-123) (Figure 4A). For both compounds the oxygen atom at carbon 3 of the sterol body (i.e. the oxygen in the OH group or acetyl ester group in lupeol or ac-LA, respectively) forms a hydrogen bond with Asn53. In the case of lupeol this oxygen atom is additionally bound by Arg25. The binding of ac-LA to Akt1 is additionally stabilized by several charged-charged and charged-to-non-charged interactions between the carboxyl group of ac-LA and the amino acids Glu17, Tyr18 and Arg23. In addition, hydrophobic interactions of the sterol scaffolds with the surrounding amino acids Phe55, Leu52, and Ile84 (Ile84 only for ac-LA) were observed.

The binding energy of ac-LA for the PH domains of Akt1 and Akt3 was similar, whereas ac-LA was not predicted to form stable complexes with the PH domain of Akt2 (binding free energy > 0). In addition to the PH domain, ac-LA was predicted to bind to the ATP binding site of the Akt catalytic domain. The binding free energy of lupeol to either PH domain of Akt1 and Akt2 was similar, but estimated to be smaller than the ones predicted for ac-LA. Interestingly and different to ac-LA, lupeol was predicted to bind also to the Akt2 PH domain. Both, ac-LA and lupeol, fit into a similar shallow pocket in the Akt1 PH domain built mainly from positively charged residues. As ac-LA exhibits a more negative surface charge compared to lupeol (Figure 1A), the electrostatic interactions between ac-LA and the Akt1 pocket contribute to the binding affinity. For comparison, we have performed *in silico* docking with the Akt inhibitor VIII [25], which showed similar binding potency to all three Akt isoforms, whereas ac-LA bound Akt1 and Akt3 more efficiently than Akt2 (Figure 4A).

The *in silico* data were confirmed by *in vitro* kinase assays using the three human recombinant Akt isoforms and GSK-3β as substrate. Ac-LA inhibited the kinase activity of all three isoforms in the following order of efficacy Akt1 > Akt3 > Akt2. Compared to ac-LA, lupeol was about threefold less potent in the inhibition of Akt1 and Akt3 isoforms, but was equally potent in inhibiting Akt2 (Figure 4B). Thus, the relative potencies of ac-LA and lupeol correlated well to their calculated affinities to Akt1-3 PH domains (Figure 4A). This indicated that the respective binding affinities to the Akt PH domain might largely govern the efficacies of ac-LA and, to a lesser extent, lupeol on the Akt signaling pathway.

To prove this hypothesis, we have used a human recombinant Akt1 construct lacking the PH domain (Akt1ΔPH). Akt1ΔPH was resistant to lupeol (≤ 30 μM) and to Akt inhibitor III (Akt-I-1/2, 10 μM), a PH domain-dependent allosteric Akt inhibitor [25]. By contrast, the inhibitory efficacy of ac-LA was strongly reduced, but not totally abolished, when Akt1ΔPH was used (Figure 4C). This indicates, that binding to the Akt PH domain might not be the sole determinant of the ac-LA inhibitory activity, and at high concentrations, ac-LA might also inhibit Akt through binding to the ATP binding site, which has been predicted by the docking approach.

### Ac-LA inhibits growth of prostate cancer xenografts and angiogenesis

**Figure 5:**
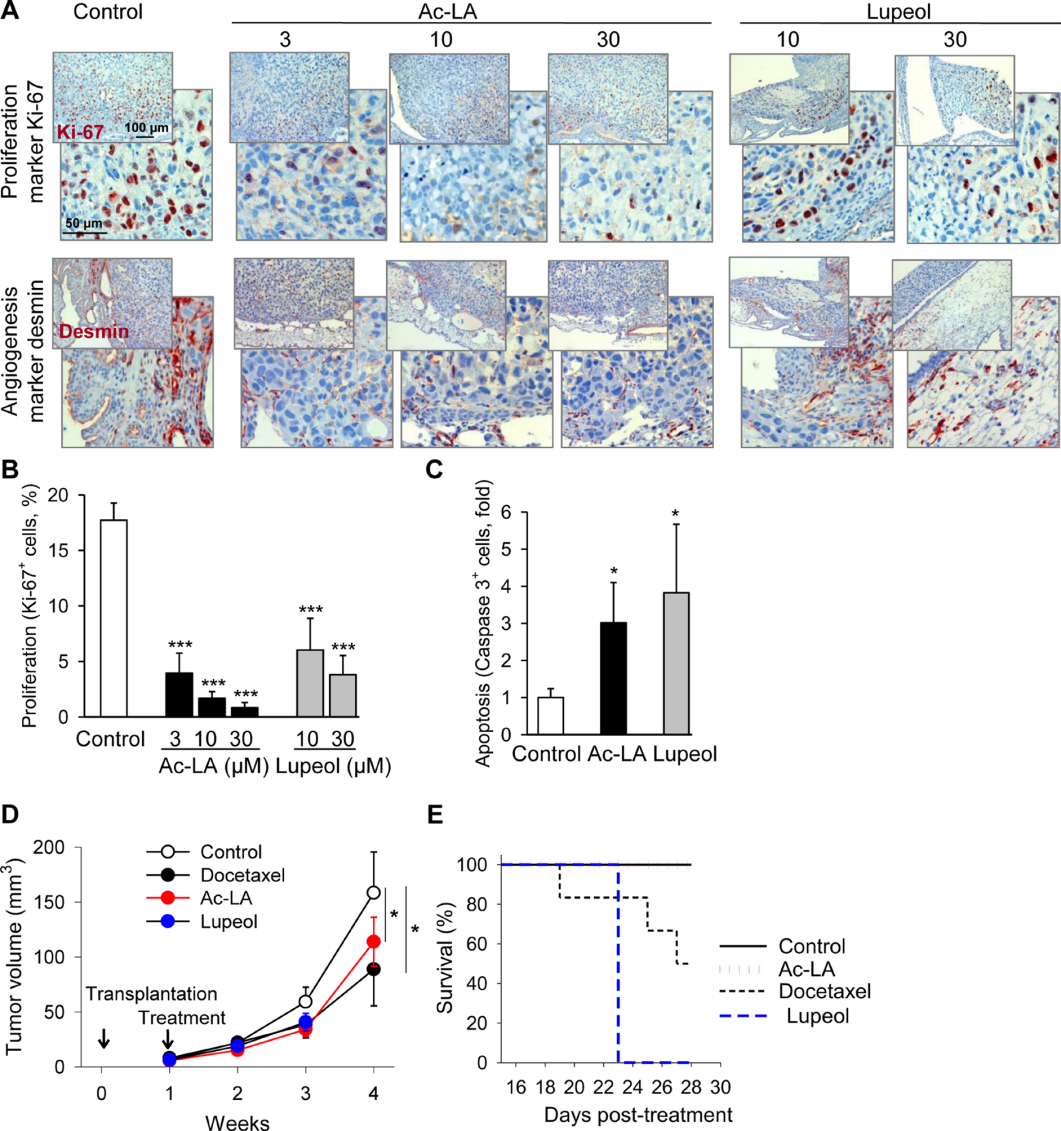
Ac-LA inhibits cell proliferation *in vivo* and angiogenesis and induces apoptosis in prostate cancer xenografts (**A**–**C**) PC-3-xenografts topically treated with ac-LA or lupeol decrease expression of the proliferation antigen Ki-67 and the angiogenesis marker desmin. One day after grafting on CAM of fertilized chick eggs, PC-3 xenografts were treated with either ac-LA or lupeol for 3 consecutive days. On day 5 after grafting, tumors were collected and analyzed immunohistochemically. Ki-67^+^ cells - brown stained nuclei, desmin - red cellular stain. Data shown are representative of four eggs. Original magnification 200x. (B) Histomorphometric analysis in sections of tumor xenografts shown in (A) Quantification of cell proliferation (Ki-67^+^ cells/high power field). (C) Histomorphometric analysis in sections of tumor xenografts shown in A. Quantification of apoptosis (caspase 3^+^ cells/high power field). Results are mean ± SEM, *p < 0.05, ***p < 0.001, *n* = 4. (**D**) Inhibition of prostate tumor growth by ac-LA and lupeol (each at 100 μmol/kg) in mice. Animals were treated intraperitoneally starting from day 8 after xenotransplantation. Docetaxel (10 μmol/kg) was used as positive control. (**E**) Kaplan-Meier graph of mouse survival shows better safety of ac-LA compared to docetaxel and lupeol. Data are mean ± SEM, *p < 0.05, *n* = 6 mice in each group.

To verify the proapoptotic and antitumor activity of ac-LA *in vivo*, prostate cancer xenografts grown on chorioallantoic membranes of fertilized chick eggs (CAM) [5, 18] were used to investigate the effects of either ac-LA or lupeol on tumor growth. Immunohistochemical analysis of xenografts revealed significant concentration-dependent inhibitory effects of ac-LA and, to a lesser extent, also lupeol on cancer cell proliferation as analyzed by expression of the proliferation antigen Ki-67 (Figure 5A, 5B). In agreement with the reduced production of the angiogenic VEGF by ac-LA *in vitro* (Figure 3D), expression of the angiogenesis marker desmin was significantly reduced *in vivo* as well (Figure 5A). Lupeol had little, if any effect on angiogenesis *in vivo* (Figure 5A), and similarly, did not inhibit the VEGF release by PC-3 cells *in vitro* (Figure 3D). In addition, ac-LA and lupeol increased the proportion of cells expressing active caspase 3, thus inducing apoptosis in prostate cancer xenografts *in vivo* (Figure 5C).

In mice, ac-LA significantly retarded growth of pre-established prostate tumor xenografts (Figure 5D). Ac-LA exhibited efficacy similar to docetaxel; it was however, much safer. All mice survived 3 weeks of daily i.p. treatment with ac-LA without any overt systemic toxicity. In contrast, mice did not tolerate the equivalent i.p. dose of lupeol for more than 2 weeks, and only 50% of the mice survived a 3-week treatment with docetaxel (Figure 5E). Weight loss and heavy diarrhea not compatible with survival were the most prominent manifestations of the lupeol-induced gastrointestinal and hepatic toxicity. Docetaxel-treated mice suffered from diarrhea and changes in plasma liver enzyme values, but they exhibited no weight loss. Neither of those adverse effects were displayed by mice treated with ac-LA.

## DISCUSSION

Within the last decades, the rate of deaths from prostate cancer decreased due to its early diagnosis by the screening for prostate-specific antigen (PSA). However, prostate cancer still ranks second among cancer-related deaths as available drugs often fail in advanced cancer stages [1].

The majority of high-grade prostate cancer and prostatic intraepithelial neoplasias exhibit overexpression of activated Akt. There is a significant correlation between the loss of the tumor suppressor gene PTEN, a negative regulator of Akt, and prostate cancer grade, chemoresistance, and relapse after therapy [26]. The PC-3 prostate cancer cell line used in this current study also harbors a PTEN-null mutation resulting in constitutive activation of Akt and its downstream target mTOR [1, 3].

At present, docetaxel (Taxotere^®^) has proven to be effective against a wide spectrum of clinical tumors including also pancreatic cancer and it is the standard chemotherapy for hormone-refractory prostate cancer [1, 2]. The taxane docetaxel promotes β-tubulin polymerization and consequently disrupts microtubule dynamics. Docetaxel leads to cell cycle arrest in G_2_/M phase, to mitotic catastrophe, and activation of apoptosis [27]. Prostate cancer cells, however, develop resistance to docetaxel. The PI3K/Akt/mTOR pathway is related to development of resistance to docetaxel in prostate and other cancers [1, 3]. Likewise, Akt overactivation has been implicated in resistance development to many conventional therapies, including the most promising ones acting at the level of growth factor receptors and mTORC1. Hence, there is hope to overcome existing chemotherapy resistance with the help of Akt inhibitors [3, 28]. Yet the optimal approaches and the optimal inhibitors still remain to be identified.

Most Akt inhibitors described in the literature compete with ATP for the binding site and have, therefore, poor selectivity for one Akt isoform against another and for related kinases [28]. In fact, clinical trials with Akt inhibitors binding to the ATP binding site were disappointing. A combinatory regimen of perifosine with bortezomib and dexamethasone failed to yield a benefit in progression-free survival and was therefore discontinued. High toxicity and low response were also observed with the ATP-competitive pan-Akt inhibitor afuresertib [3]. These disappointing results can be explained by high structural similarity of the ATP pocket among serine/threonine kinases [28].

Considering those difficulties, a number of Akt inhibitors targeting the PH domain have been developed. The purine analogue triciribine is one of the best-known PH-dependent inhibitors of Akt in clinical studies. Unfortunately, the monotherapy trials with triciribine of tumors characterized by high Akt activation were not further pursued as they revealed insufficient antitumor activity besides high incidences of hepatotoxicity, hypertriglyceridemia, thrombocytopenia, and hyperglycemia as adverse effects [28].

Our data demonstrate that ac-LA-induced inhibition of Akt depends largely and that of lupeol fully on the presence of the PH domain. *In silico* modeling confirmed that compared to lupeol, ac-LA has a stronger binding affinity to the Akt1 PH domain *via* hydrogen bonding interaction with residues Glu17, Tyr18, Arg23, and Asn53. Remarkably, the same residues, which are involved in the binding of ac-LA have been shown to be important for the binding of the lipid head of phosphoinositides (Glu17, Arg23, and Asn53) [29]. Within the same shallow binding pocket, lupeol is engaged with only one hydrogen bond to Arg25, which is essential for the Akt activation by phosphoinositides.

The recent elucidation of the crystal structures of Akt1 and Akt2 with the Akt inhibitor VIII has shown that the PH and kinase domains cooperate to upregulate Akt activation [30]. Because *in silico* docking indicates stronger binding affinity of ac-LA to full length Akt1, ac-LA might bind to the pocket formed by the PH and kinase domains, similarly to that described for the allosteric Akt inhibitor VIII [30]. However, one should note that Akt inhibitor VIII depends entirely on the PH domain, whereas ac-LA still inhibits Akt1ΔPH, although to a lesser extent. A derivative of the inhibitor VIII, MK-2206, displayed a marked synergistic effect with several chemotherapeutic agents in animal studies and is in clinical trials [28]. A modest clinical benefit in breast cancer patients prompted a new clinical trial with patients with breast cancer harboring mutations leading to Akt overactivation [3]. The drug, however, exhibits high toxicity, has poor pharmacokinetics, and requires quadruple applications per day [3, 28].

Ac-LA is stable and well tolerated by animals. In addition, it discriminates between the Akt isoforms. This feature might be beneficial in clinical settings, where strong inhibition of Akt2 might lead to insulin resistance [3]. Thus, different to other inhibitors such as afuresertib, AZD5363, GDC-0068, GSK690693, and AT7867, as well as the indirect Akt inhibitors PX-866 and perifosine [3] and lupeol, ac-LA preferentially inhibits Akt1 over Akt3 and Akt2. There is a good correlation between cancer cell toxicity and Akt1 inhibition by ac-LA in the μM range.

Akt is not the only kinase to contain a PH domain, which is activated by phosphoinositide binding. Our data show that ac-LA inhibits also the PDK1 activation, which as well relies on the PH domain for transfer to biological membranes. Therefore, it is feasible that Akt is not the only intracellular target of ac-LA. Hence, inhibition of PDK1, which is constitutively active and is capable of activating a number of AGC kinases (including Akt, S6K, SGK, PKC, PKN, and others), might ensure additional repression of cancer-relevant Akt-independent pathways in cancer cells [1]. However, as lupeol and ac-LA inhibit PDK1 to a similar extent, the more favorable pharmacodynamic efficacy of ac-LA might be independent of the PDK1 activation.

Compared to lupeol, ac-LA exhibited far better cytotoxicity against chemoresistant prostate and pancreatic cancer cells *in vitro*. Remarkably, in pre-established androgen-independent prostate tumor models, ac-LA significantly slowed the growth of tumor xenografts without overt toxicity. At variance, experiments in the lupeol group had to be discontinued after 2 weeks of treatment due to high toxicity in the applied dose. High toxicity of lupeol has not been recognized earlier. It has been shown that oral and topical administrations of lupeol were well tolerated. Intraperitoneal administration of lupeol complexed by γ-cyclodextrin, however, resulted in high cytotoxicity, which should be considered in future studies. Differently to lupeol and docetaxel, ac-LA affected neither liver blood values, the gastrointestinal tract, nor the animal weight, nor did it exhibit any overt systemic toxicity during 3 weeks of intraperitoneal administration.

Another interesting difference between ac-LA and lupeol concerns their different effects on the VEGF release by prostate cancer cells *in vitro* and likewise angiogenesis *in vivo*. Expression of different angiogenic factors including VEGF or PDGF is controlled by hypoxia-inducible transcription factor 1α. Its translation is induced, in turn, by a master translation regulator, the mTORC1 substrate, P70S6K [4]. Ac-LA strongly reduced the VEGF release by PC-3 cells *in vitro* and angiogenesis in prostate cancer xenografts *in vivo*. Hence, the reduction of VEGF-levels by ac-LA is in accordance with the inhibitory effect on Akt and its downstream targets. Differently, lupeol did not inhibit the phosphorylation of the mTORC1 substrate P70S6K, rather the P70S6K phosphorylation was increased two hours after treatment. Accordingly, lupeol slightly elevated the VEGF secretion by prostate cancer cells. Similarly, lupeol did not inhibit angiogenesis in prostate cancer xenografts on the CAM. At rather high doses, lupeol has been shown to inhibit angiogenesis [31], possibly as a consequence of overall toxicity.

We show here that ac-LA inhibits viability of a panel of cancer cell lines, but not that of MDA-MB-231, which carries wild type PTEN and exhibits low level of Akt pathway activation. Indeed, among 11 cell lines tested, MDA-MB-231 exhibited the lowest level of the Akt pathway activation (NCI database, https://dtp.cancer.gov). Thus, the relative levels of the phosphorylated forms of GSK-3α and GSK-3β, as well as those of Akt-Thr308 and Akt-Ser475 are 0.56, 0.07, 0.53, respectively, whereas the same values for PC-3 cells are 0.71, 0.14, 1.70, respectively. Of note, Akt inactivation in MDA-MB-231 leads to disruption of the Akt-PLCγ interaction and accelerates the cell accumulation at the G_2_/M checkpoint [32]. Indeed, both, ac-LA and lupeol, increased the number of MDA-MB-231 cell in the G_2_/M phase, but induced no apoptosis in these cells. These data are likewise in accordance with findings showing high resistance of MDA-MB-231 cells to PI3K inhibitors probably due to K-Ras mutations [33], which contribute to the S6K phosphorylation through the MEK-ERK pathway bypassing Akt. Thus, MDA-MB-231 possesses antiapoptotic mechanisms to avoid the ac-LA- and the lupeol-induced apoptosis.

Taken together, the Akt inhibitor ac-LA is a promising compound for potential cancer therapy owing to its stability, low systemic toxicity, and multifaceted effects on the entire signaling network of cancer cells. Ac-LA is superior to lupeol, based on more potent antiproliferative, antitumorigenic, antiangiogenic, and on apoptosis-inducing effects, as well as the absence of adverse effects in mice. The pharmacological properties of ac-LA and lupeol differ in favor of ac-LA. Favorable pharmacokinetic and pharmacodynamics properties of ac-LA in the absence of detectable intracellular conversion of ac-LA to lupeol are valuable prerequisites for its further exploration as a putative antitumor drug.

## MATERIALS AND METHODS

### Reagents

3α-acetyl-lup-20(29)-en-24-oic acid (acetyl- lupeolic acid, ac-LA) was isolated from African frankincense oleogum resin obtained from *Boswellia carterii* and purified to chemical homogeneity (> 99% purity) using reverse-phase HPLC. Ac-LA was characterized by mass spectrometry and nuclear magnetic resonance spectroscopy [15, 16, 22]. Lupeol (purity > 99%) was purchased from Carl Roth (Karlsruhe, Germany), docetaxel was from Fluka (Buchs, Switzerland), and Akt inhibitor VIII (Akt Inh.) was from Calbiochem (San Diego, CA). Lupeol was dissolved in warm alcohol and diluted 1:7.7 in dimethyl sulfoxide (DMSO). The other compounds were dissolved in pure water-free DMSO. The final concentration of solvent in medium for the control and the compounds for all treatment protocols was 0.5%.

For treatment of *in vivo* xenografts on the CAM, compounds and polyvinylpyrrolidone K10 (PVP) (Sigma), both separately dissolved in methanol, were mixed for 10 min at room temperature in a molar ratio of 1:4. Methanol was dried off at 60°C under a stream of nitrogen. The residue was lyophilized and dissolved by sonification for 2 min in 0.9% NaCl. Formation of such complexes allows the *in vivo* administration of lipophilic compounds in aqueous solutions. The microemulsion remained stable at room temperature for at least 3 days. For xenograft treatment in mice, the complexes were formed as described above but instead of PVP, γ-cyclodextrin (CD) (ISP, Wayne, NJ) was used in a ratio of 1:21 (w/w) for ac-LA/γ-CD and lupeol/γ-CD and 1:101 (w/w) for docetaxel/γ-CD.

### Cell culture

The prostate cancer cell line PC-3 (ECACC, Salisbury, UK) was maintained in F12K supplemented with 10% fetal bovine serum (FBS) and penicillin/streptomycin. The prostate epithelial cell line RWPE-1 (ATCC, Manassas, VA) was cultivated in keratinocyte serum-free medium supplemented with 5 ng/ml EGF, 0.05 mg/ml bovine pituitary extract, and penicillin/streptomycin. The pancreatic cancer cell line MIA-PaCa-2 (ATCC, Manassas, VA) was maintained in DMEM with 10% FBS and penicillin/streptomycin. All other cell lines were from ATCC and were cultured under standard conditions as recommended by the supplier.

### Quantification of compounds by reverse-phase HPLC

Treated cells were scraped, washed with PBS and lysed in MeOH/H_2_O/AcOH (90:10:0.2, vol/vol/vol). The filtrated lysates were analyzed by reverse-phase HPLC using ac-LA and lupeol as reference standards (Büchele et al. 2003). Intracellular concentrations were calculated using an average PC-3 cell diameter of 18.1 μm as reported for PC-3 in the NCI-60 cell panel by Nexcelom Bioscience (Lawrence, MA, http://www.nexcelom.com) yielding a single-cell volume of 3,100 fl.

### Cell viability and apoptosis analysis

Cellular viability was analyzed by colorimetric quantification of reduced XTT as a criterion for mitochondrial activity. XTT sodium salt (0.2 mg/ml) and phenazine methosulfate (PMS) (1.5 μg/ml), both from Sigma, were given to treated cells and the spectrophotometrical absorbance at 450 nm was measured using a microtiter plate reader. Control samples were treated with solvent. For quantification, a medium blank was subtracted from the samples and the absorbance was normalized to control. Besides, early apoptotic cells exhibiting expression of phosphatidylserine on the outer leaflet of the cell membrane were analyzed by flow cytometry after staining for 15 min with fluorescein isothiocyanate-labeled annexin V (BD Pharmingen, San Diego, CA). Late apoptotic cells were excluded based on PI (Sigma) binding to DNA. Specific early apoptosis was calculated as the percentage of annexin V^+^/PI^-^ cells.

Additionally, annexin V/PI-stained PC-3 cells were detected using spinning disk confocal fluorescence microscopy (Carl Zeiss, Jena, Germany) [34]. Cells were seeded in a μ-Slide VI^0.4^ from IBIDI (Martinsried, Germany), treated for 24 h with compounds, stained with FITC-labeled annexin V and PI, and acquired using green and red filters.

Reduced DNA content in the apoptotic sub-G_1_-population of the PI-stained PC-3 cells was analyzed flow cytometrically. DNA-fragmentation of apoptotic PC-3 cells was visualized by agarose gel electrophoresis. DNA was extracted from PC-3 cells treated for 48 h following instructions of the manufacturer (Boehringer, Mannheim, Germany). DNA was visualized with ethidium bromide.

Activity of caspase-3 was measured in cells treated with ac-LA or lupeol for 6 h and incubated with the caspase 3 substrate, Z-DEVD-rhodamine 110 (100 μM, Invitrogen, Carlsbad, CA) by using flow cytometry (FACSVerse, BD, Heidelberg, Germany). Mitochondrial membrane potential was likewise analyzed in cells treated with either ac-LA or lupeol (both 10 μM) for 6 h, stained with JC-1 (Molecular Probes) followed by flow cytometric analysis [21, 35].

### VEGF ELISA

Active VEGF in the supernatant of PC-3 cells was detected by enzyme-linked immunosorbent assay following instructions of R&D Systems (Minneapolis, MN). Absorption was measured with a microplate reader at 450 nm using a reference wavelength of 570 nm. Chetomin (100 nM, Cayman, Ann Arbor, MI), a HIF-signaling inhibitor, was used as positive control.

### Akt kinase assays

Kinase assays were performed as described previously [18, 36]. Active recombinant human kinases Akt1, Akt2, and Akt3 were from Biaffin (Kassel, Germany), Akt1ΔPH (S473D) was from Millipore (Dundee, UK). GST-fused recombinant GSK-3β (Cell Signaling, Danvers, MA) was used as substrate. Kinases were preincubated for 15 min at 30°C with ac-LA, lupeol, 10 μM Akt inhibitor VIII, or DMSO as solvent control before substrate and [γ-^32^P]ATP were added. After 20 min at 30°C, proteins were separated using SDS-polyacrylamide gel electrophoresis. After drying, the GSK-3β phosphorylation was visualized using a PhosphorImager (GE Healthcare, Buckinghamshire, UK).

### Western immunoblotting

Equal amounts of proteins of whole cell lysates or nuclear extracts [36] were separated by SDS-polyacrylamide gel electrophoresis. Proteins were transferred to a polyvinylidene difluoride (PVD) membrane, probed with specific antibodies, and detected using ECL detection reagent (GE Healthcare).

Antibodies against p-PDK1^S241^ (#3031), p-GSK-3β^S9^ (#9336), p-BAD^S136^ (#39295), AKT1 (#2967), and β-catenin (#9562) were from Cell Signaling. P-Akt1^S473^ (#05-736) antibody was from Upstate (Charlottesville, VA), p65 (1546-1), p-Akt1^T308^ (#2214-1), and p-P70S6K^T389^ (#1175-1) were supplied by Epitomics (Burlingame, CA), and c-myc (M5546) was from Sigma. The antibodies against IκBα (sc-371), P70S6K (sc-8418), BAD (sc-8044), GSK-3 (sc-7291), and topoisomerase I (TOPO I) (sc-10783) were from Santa Cruz Biotechnology (Santa Cruz, CA), PDK1 (IMG-30048) was from Imgenex (San Diego, CA), and actin (MAB1501) was from Chemicon (Temecula, CA).

### Molecular modelling and docking

2D structures of ac-LA, lupeol, and Akt inhibitor VIII were generated using Accelrys Discovery Studio 3.5 (Accelrys Inc., San Diego, CA) and were converted to 3D structures by Ligprep 3.5 module in Schrödinger suite 2015. Structures were optimized using OPLS-2005 (Optimized Potentials for Liquid Simulations) force field and were subjected to ionization, stereoisomer generation, as well as energy minimization.

For surface charge distribution, the quantum chemistry calculation was performed by Gaussian 09 using 6-31G basic set and the Restrained Electrostatic Potential (RESP) charge was generated by Antechamber in AmberTools 16 and visualized by Maestro 10.4 in Schrödinger suite 2015 (Schrödinger, LLC, New York, NY). Atoms with negative partial charge were colored red, while the atoms with positive partial charge were colored blue.

The X-ray structure of Akt1 (PDB ID:1H10), Akt2 (PDB ID: 1P6S), Akt3 (PDB ID: 2×18) PH domains and Akt kinase domain (PDB ID: 2×39) were used for docking experiments. High resolution structure of the PH domain of Akt bound to Ins(1,3,4,5)-tetrakisphophate (Ins(1,3,4,5)P_4_, PDB: 1H10) served as a model. Hence, the atomic coordinates of the Akt1/Ins(1,3,4,5)P_4_ complex were processed with Protein Preparation Wizard using Schrödinger suite 2015 as following: missing hydrogen atoms were added, OPLS-2005 force field was assigned, water and Ins(1,3,4,5)P_4_ removed, and restrained minimization applied.

Semi-flexible and flexible (induced) fit docking were used to predict the possible binding position of ac-LA and lupeol to the Akt1 PH domain (Schrödinger suite 2015). Rigid ligand docking and ligand free energy prediction were performed using the Glide module and a 72 × 72 x 72 Å grid box (active centre), which included the Ins(1,3,4,5)P_4_ binding site, and a docking protocol running in SP mode. The flexible docking, which considers ligand flexibility, was performed using the Induced Fit Docking (IFD) wizard of Schrödinger suite 2015. Complexes obtained using this protocol were further refined using Prime program (Schrödinger suite 2015), and the binding affinity was calculated with Gscore. The best docking model was visualized using PyMOL 1.3 (Schrödinger suite 2015).

### *In vivo* experimental design

For PC-3-xenografts grown on CAM, 0.65 × 10^6^ PC-3 cells in 20 μl of medium/matrigel (BD Biosciences, San Jose, CA) (1:1) were seeded on CAM of fertilized chick eggs incubated at 37°C for 8 days. A day later, pre-established xenografts were topically treated with either ac-LA/PVP, lupeol/PVP, or PVP vesicles once daily for 3 days. On day 4 after the onset of treatment, xenografts were fixed, paraffin-embedded, serially cut (thickness of 5 μm), and mounted onto glass slides. The sections were stained with antibodies against Ki-67 proliferation antigen (M7240), desmin angiogenesis antigen (M076001) (both from Dako Corp., Glostrup, Denmark), and counterstained with hematoxylin. Alternatively, sections were stained with fluorescence-labeled antibodies against active caspase-3 (Ab2302), Biozol, Eching, Germany) and DAPI nuclear stain (Roche, Basel, Switzerland). Images of the sections were digitally recorded using Visupac 22.1 software with an Axiophot microscope (both from Carl Zeiss, Göttingen, Germany) and a MA-3249 CCD camera (Sony, Tokyo, Japan). Photomicrogaphs were digitally analyzed with ImageJ 1.43m software (NIH) using the respective color thresholds. Pixel areas of corresponding positive cells were normalized to the area of all tumor cells as obtained from hematoxylin or DAPI staining.

For xenotransplantation in mice, 7–8 weeks old male NMRI/nu-nu mice (Janvier, Le Genest-St.-Isle, France) were inoculated with 0.5 × 10^6^ PC-3 cells in 0.1 ml medium/injection in the subscapular region at both dorsal sides. After 1 week, pre-established xenografts were treated once daily by intraperitoneal injection of 0.2 ml ac-LA-γ-CD (100 μmol/kg), lupeol-γ-CD (100 μmol/kg), or equivalent γ-CD vesicles. Docetaxel-γ-CD (10 μmol/kg, 8 mg/kg) was applied once per week due to its higher toxicity [27]. Tumor volume was calculated according to the formula 0.5 × length × width × thickness [18].

The animal experiments have been reviewed and permitted by the state authorities and were therefore conducted according to the relevant national and international guidelines.

### Statistical analysis

Quantitative results show the mean ± SEM. As indicated, statistical significance was analyzed using multigroup comparisons and the Newman-Keuls or Tukey's tests with the software package Statistica 7.0.
